# Autosomal mutations affecting Y chromosome loops in *Drosophila melanogaster*

**DOI:** 10.1186/1471-2156-9-32

**Published:** 2008-04-11

**Authors:** Francesca Ceprani, Grazia D Raffa, Romano Petrucci, Roberto Piergentili

**Affiliations:** 1 Dipartimento di Genetica e Biologia Molecolare, Sapienza – Università di Roma, Piazzale Aldo Moro 5, 00185 Rome, Italy

## Abstract

**Background:**

The Y chromosome of *Drosophila melanogaster *harbors several genes required for male fertility. The genes for these fertility factors are very large in size and contain conspicuous amounts of repetitive DNA and transposons. Three of these loci (*ks-1*, *kl-3 *and *kl-5*) have the ability to develop giant lampbrush-like loops in primary spermatocytes, a cytological manifestation of their active state in these cells. Y-loops bind a number of non-Y encoded proteins, but the mechanisms regulating their development and their specific functions are still to be elucidated.

**Results:**

Here we report the results of a screen of 726 male sterile lines to identify novel autosomal genes controlling Y-loop function. We analyzed mutant testis preparations both *in vivo *and by immunofluorescence using antibodies directed against Y-loop-associated proteins. This screen enabled us to isolate 17 mutations at 15 loci whose wild-type function is required for proper Y-loop morphogenesis. Six of these loci are likely to specifically control loop development, while the others display pleiotropic effects on both loops and meiotic processes such as spermiogenesis, sperm development and maturation. We also determined the map position of the mutations affecting exclusively Y-loop morphology.

**Conclusion:**

Our cytological screening permitted us to identify novel genetic functions required for male spermatogenesis, some of which show pleiotropic effects. Analysis of these mutations also shows that loop development can be uncoupled from meiosis progression. These data represent a useful framework for the characterization of Y-loop development at a molecular level and for the study of the genetic control of heterochromatin.

## Background

Notwithstanding the recent advances in genomics, mainly thanks to the completion of model organisms DNA sequencing, there is still a part of the eukaryote genome which is largely unknown in both structure and function: the heterochromatin. Heterochromatin is a complex of DNA and specifically associated proteins, is characterized by low gene density and the presence of highly repetitive sequences, and accounts for an important portion of the genome in all organisms. For several decades it has been considered as the repository of the so-called 'junk DNA', characterized by several selfish sequences whose only function seems that of reproducing themselves from one generation to the next. For a long time, the only exceptions were represented by the centromeres and telomeres, which are important elements for chromosome stability and proper segregation during cell division. Later studies demonstrated that moving a euchromatic gene next to a heterochromatic region causes its silencing, a phenomenon known as position effect variegation (PEV, see [1] for review). This indicates that the expression of a gene can be influenced by placing it in a heterochromatic context. Moreover, heterochromatin contains functional protein-encoding genes (see [2] for review), often larger than the average euchromatic gene since they usually have very long introns [3,4]. Interestingly, the expression of heterochromatic genes is not properly regulated if the structure of the surrounding heterochromatin is altered [5,6]. However, the nature of heterochromatin, its biological function and the reason why it is so abundant are still topics under investigation, and the study of its DNA content is still at a preliminary stage [7,8].

One of the largest clusters of heterochromatin resides in the Y chromosome of most animals. The Y chromosome of *Homo sapiens *is ~37.5 Mb long and 95% of the chromosomal DNA is Y-specific, with no homology to the X chromosome [9,10]. In this regard the *Drosophila melanogaster *Y chromosome is quite similar: its DNA content is ~40 Mb and mostly Y-specific, with the exception of the nucleolar organizer [11]. In 1916 Bridges [12] demonstrated that this chromosome is not required for viability; flies with an X/0 karyotype are phenotypically male, but they are completely sterile. This indicates that the Y chromosome carries genes required only for male fertility. In 1960 Brosseau [13] mapped at least 6 genetic loci on it, each of which spanning several thousand kilobases of DNA, as demonstrated later [14-16]. These 'fertility genes' play a role only in the male germ line [17], specifically in primary spermatocytes (see [18] for review). Their length is ~4 Mb, more than 100 times larger than the average eukaryotic gene. Three fertility factors, namely *kl-5 *and *kl-3 *on the long arm and *ks-1 *on the short arm [16] assemble prominent lampbrush-like loops in primary spermatocytes nuclei, representing the cytological manifestation of their activity [19]. The kl-5 and ks-1 loops appear darker when viewed using phase contrast optics, although they probably have a thread-like molecular organization [20]. The kl-3 loop is composed of a thinner filament and shows a more diffuse appearance. Loop development in primary spermatocytes is strictly controlled and sequential: kl-5 and ks-1 develop before kl-3 during spermatocytes growth; all of which subsequently disintegrate during meiotic prophase I [19]. One major characteristic for all loops is that they are bound by several proteins, which determines their cytological appearance. In the past, various antibodies directed against loop-binding proteins have been raised. These proteins represent non-Y encoded antigens including DNA-interacting proteins [21], RNA-interacting proteins [19,22-26] and testis-specific antigens that are incorporated either in nuclei [27] or in sperm tails [19,28-31] during late stages of spermiogenesis.

In the present work we have screened 726 autosomal male sterile lines from four different collections, for Y-loop alterations. In order to characterize the presence and morphology of Y-loops in these mutants, we have utilized two antibodies directed against loop-binding proteins. The first is the S5 antibody that recognizes a 70 kD protein known to be associated to nascent RNAs [22,32]. This antibody produces an intense staining of the kl-5 loop and a weaker staining of the ks-1 loop, but shows no cross reaction with the kl-3 loop [19,23]. The second is the T53-1 antibody that reacts with a testis specific axonemal component related to leucine aminopeptidases [33,34], which accumulates on the kl-3 loop only [29]. Cytological and genetic analysis of all mutants permitted us to isolate 17 mutations at 15 loci that affect the normal behavior of loop-forming fertility factors in primary spermatocytes. Four of these loci seem to have a specific role in loop formation and control, since no other defect but aberrant loops and immotile sperm were detected using our experimental conditions. Other mutations displaying pleiotropic effects are also discussed.

## Results

### Screening of male sterile collections

In order to identify mutations affecting loop development we screened four different collections of autosomal male sterile mutants (Table 1). The Hackstein collection consists of 23 male sterile lines showing alterations in primary spermatocyte development. These lines had been previously selected from a larger collection of 400 male sterile mutations induced by EMS [35]. The Wasserman collection consists of 62 lines mutagenized by the mobilization of a *rosy*^+ ^P transposon [36]. Four of these lines, however, have accumulated an additional early lethal mutation on the same chromosome, so they were not analyzed further. The Ceprani collection consists of 44 male sterile lines induced in our laboratory (Ceprani, unpublished data) through the mobilization of I transposable elements [37]. We also examined 601 third chromosome mutants from a collection of 1955 autosomal male sterile mutants previously selected by Wakimoto and coworkers [38,39]. These lines derive from the Zuker collection of ~12,000 viable lines generated by EMS treatment [40]. Collectively, we obtained cytological data from 726 lines.

**Table 1:** Male sterile mutants scored for abnormal loop development

	**Hackstein**	**Wasserman**	**Ceprani**	**Zuker**	**Total**
**Lines**	**23**	**58**	**44**	**601**	**726**
**Loops only**	5	1	0	2	8
**Pleiotropic**	1	1	5	7	14
**Loci identified**	4	2	5	7	17

Summary of the results from 726 male sterile lines screened by immunofluorescence. Line *ms(3)155-34 *from Zuker collection fails to complement line *ms(3)HB223 *from Hackstein collection, resulting in 17 total identified loci.

All mutants were first analyzed *in vivo *to select for lines with immotile sperm; lines showing degeneration of most germ cells were discarded. Testes from selected lines were fixed as described by Pisano and coworkers [29] and immunostained with the S5 and the T53-1 antibodies. All lines exhibiting only weak defects in Y-loops were excluded. Our immunohistochemical analysis resulted in the isolation of 17 mutations causing defects in loop morphology and/or development. Complementation tests revealed that these mutations identify 15 loci (Table 2). The present screening permitted us to define two classes of genes whose wild-type function is implicated in controlling loop development. One class is represented by 4 loop-specific genes, identified by 5 mutations, which elicit only abnormal loops and immotile sperm. The other class consists of 11 genes, identified by 12 mutations, causing several meiotic and/or post-meiotic defects, besides abnormalities in loop development. In all mutants male sterility is due to immotile, grossly abnormal, or absent sperm.

**Table 2:** Mutations affecting loop development and their phenotypes

**Mutant line **(***gene name***)	**Source**	**Map position**	**kl-3**	**kl-5**	**ks-1**	**Spermatids**	**Sperm tails**
*ms(2)HA10 (lup-4)*	H	55D1-E6	-	+	+	-	-
*ms(2)HA30 *(*lup-4*)	H	55D1-E6	-	+	+	+	nm
*ms(2)HB108 *(*lup-3*)	H	2–46.6	-	+	+	+	nm
*ms(2)04818 (dbf)*	W	32A1-2	-	+	+	-	-
*ms(3)03817 *(*dolly-1*)	W	80A1-F9	+	-	-	+	nm
*ms(3)127-109*	Z	3	-	+	+	-	-
*ms(3)168-112*			-	+	+	-	-
*ms(3)142-46 *(*dolly-2*)	Z	43.2–50.0	-	+	+	+	nm
*ms(3)150-16*	Z	3	-	+	+	-	-
*ms(3)162-39*	Z	85D8-E13	+	+/-	-	-	-
*ms(3)165-104*	Z	3	-	-	-	-	nm
*ms(3)167-72*	Z	63C6-F7	-	+	+	-	-
*ms(2)Fra-1*	C	2	+	-	-	-	nm
*ms(2)Fra-4*	C	2	-	+	+	-	nm
*ms(2)Fra-15*	C	2	-	+	+	-	nm
*ms(2)Fra-38*	C	2	-	+	+	-	nm
*ms(2)Fra-39*	C	2	-	+	+	-	nm

Summary of mutations with loop abnormalities identified in this analysis. First column: complementation groups and the corresponding mutant lines identified in this screening. When available, the name is indicated between parentheses. Second column: source collection (H = Hackstein, W = Wasserman, Z = Zuker, C = Ceprani). Third column: statistical map or cytological map (if available). + indicates wild type phenotype; – indicates abnormal phenotype; +/- indicates weak phenotype; nm: non-motile sperm.

**Figure 1:**
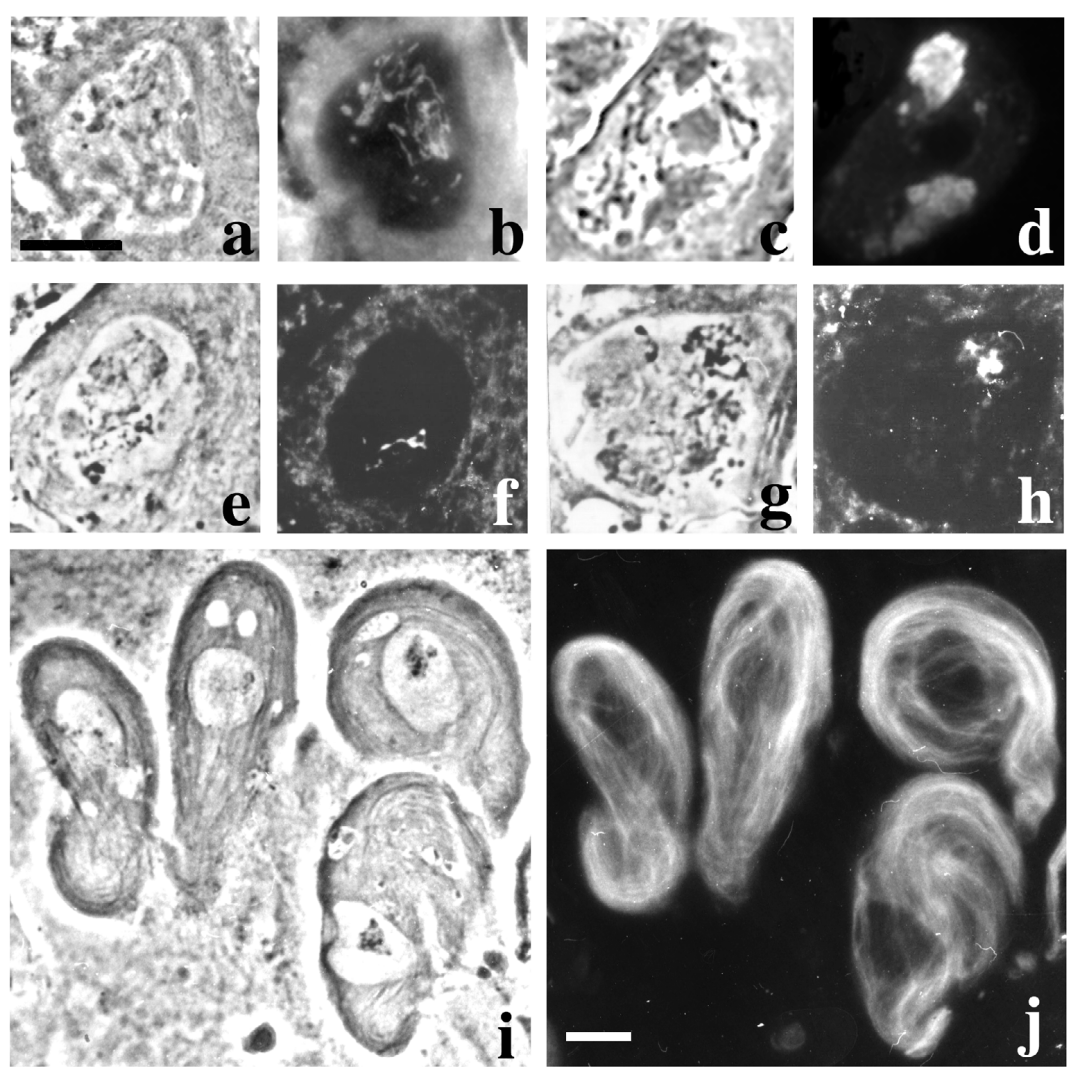
**Primary spermatocytes of mutants from Hackstein collection**. a-c-e-g-i: phase contrast; b-d-f-h-j: immunofluorescence. a-b: wild type primary spermatocyte immunostained with the T53-1 antibody; note the prominent, threadlike kl-3 loop. c-d: wild type primary spermatocyte immunostained with the S5 antibody; the brightly fluorescent loop is kl-5, the dull fluorescent loop is ks-1. e-f: *ms(2)HB108 *spermatocytes exhibit a kl-3 loop which is only partially decorated by the T53-1 antibody. g-h: kl-3 phenotype in *ms(2)HA30*; the loop appears very reduced, although not as much as in *ms(2)HB108*. i-j: *ms(2)HA10 *meiocytes immunostained with anti α-tubulin antibody. Note that sperm tails grow from spermatocyte nuclei that have failed meiosis. Bar: 10 μm

**Figure 2:**
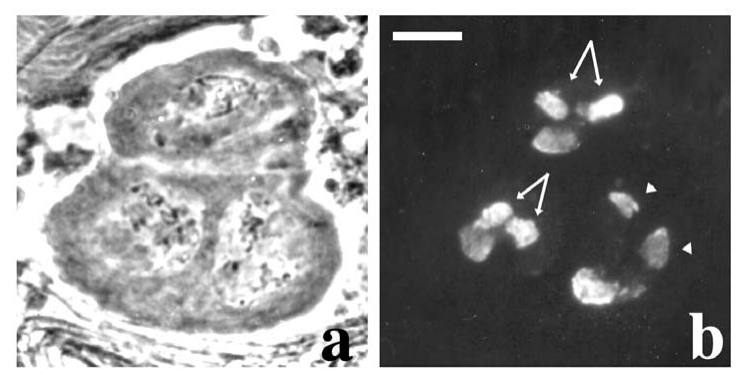
**Cytology of *dolly-1 *mutants**. a: phase contrast; b: S5 immunostaining. Note the split kl-5 (arrows) and the split ks-1 (arrowheads) loops. Bar: 10 μm

**Figure 3:**
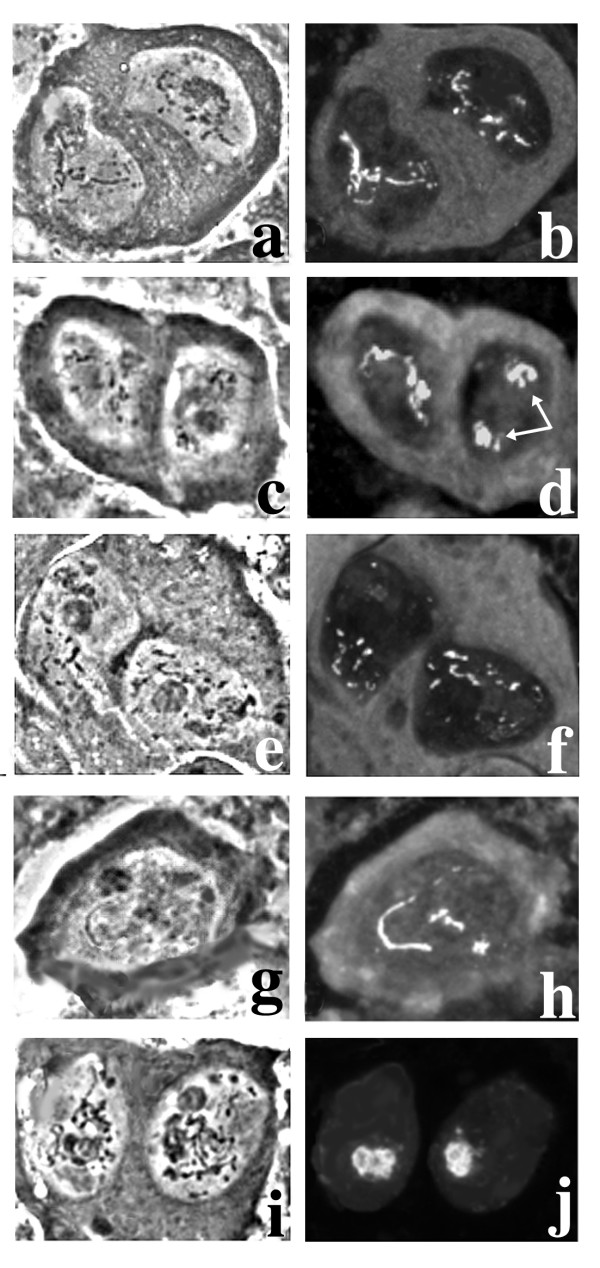
**Primary spermatocytes of mutants from Zuker collection**. Left column: phase contrast; right column: immunostaining with T53-1 (b-d-f-h) or S5 (j) antibodies. a-b: *ms(3)168-112*. c-d: *ms(3)142-46*; note the split kl-3 loop (arrows). e-f: *ms(3)150-16*. g-h: *ms(3)167-72*. i-j: *ms(3)162-39*; note that in both nuclei only the kl-5 loop reacts with the S5 antibody. See text for further explanations.

**Figure 4:**
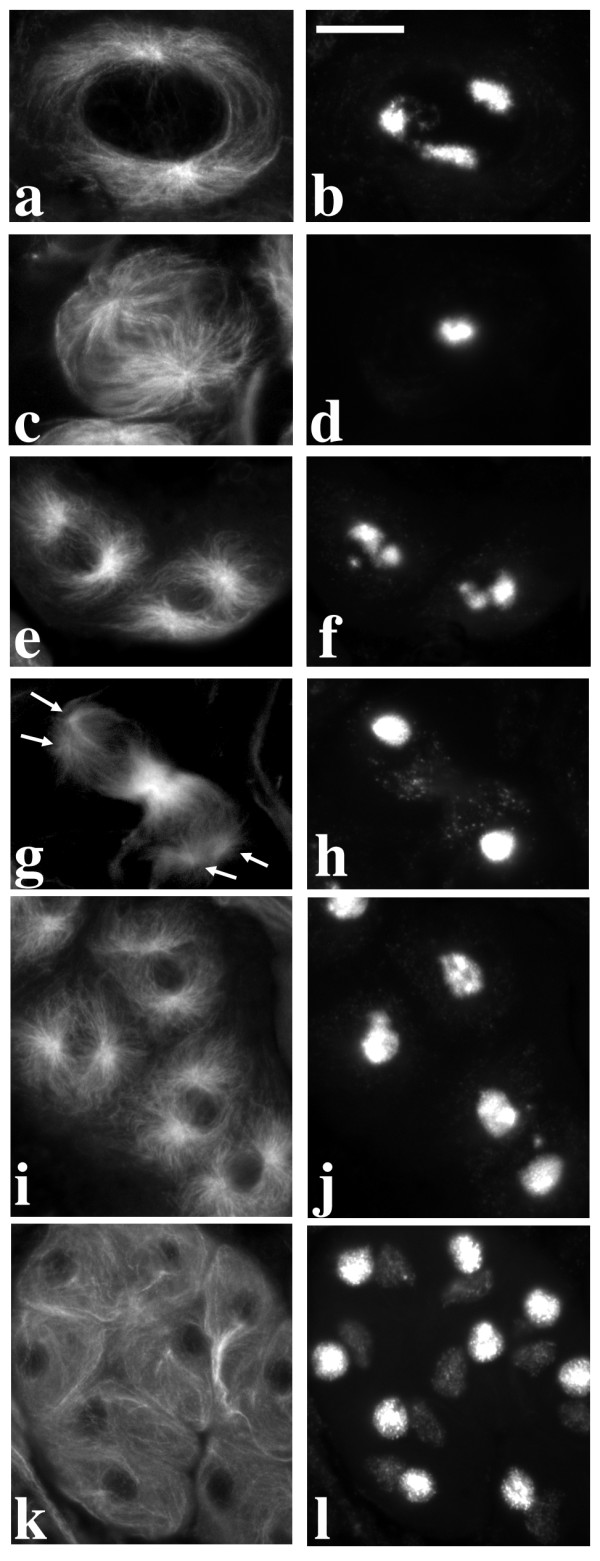
**Wild type meiosis of *D. melanogaster***. Left column: immunostaining with anti α-tubulin; right column: Hoechst 33258 staining. a-b: early prometaphase I in wild type primary spermatocytes; note the prominent asters (a) and the three clumps of chromatin (b), corresponding to the major autosomes and the sex chromosomes; spaces between the clumps are filled by Y-loops. c-d: metaphase I; chromosome condensation is complete (d), and spindle fibers contact centromeres. e-f: anaphase I. g-h: telophase I; note the replicated asters (arrows in g) and the mitochondria migrating along the spindle (small white dots in h). i-j: metaphase II cells. k-l: spermatids at the onion stage; note the weak staining of nebenkern due to mitochondrial DNA and the 1:1 association of nuclei and nebenkern of similar size. Bar: 10 μm

**Figure 5:**
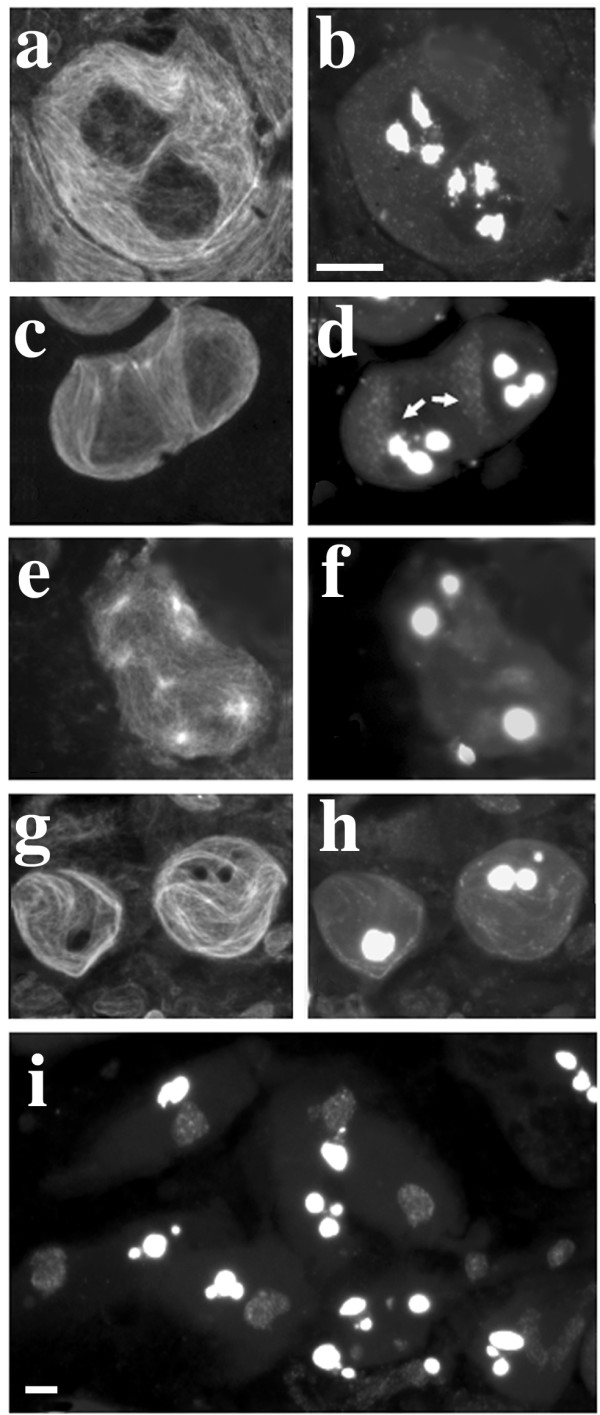
**Meiotic phenotype of *ms(3)168-112 *and *ms(3)127-109 *lines**. Left column: immunostaining with anti α-tubulin; right column: Hoechst 33258 staining. For comparison, see also the wild type meiosis reported in Figure 4. a-b: early prometaphase in primary spermatocytes of *ms(3)168-112 *mutants; chromatin is condensed (b) as in wild type but asters are absent (a). c-d: late prometaphase in *ms(3)127-109 *primary spermatocytes; arrows in (d) point to aggregated mitochondria resembling nebenkern. e-f: a meiotic cell, from *ms(3)127-109 *testes showing multiple α-tubulin nucleation foci (e) and four nuclei of different sizes (f). g-h: abnormal spermatids during the elongation stage in *ms(3)168-112 *males; note the irregular organization of microtubules (g) and the unusual chromatin distribution (h). i: spermatids from the *ms(3)168-112 *line showing irregular nuclei/nebenkern associations and irregular nuclear size. Bars: 10 μm

**Figure 6:**
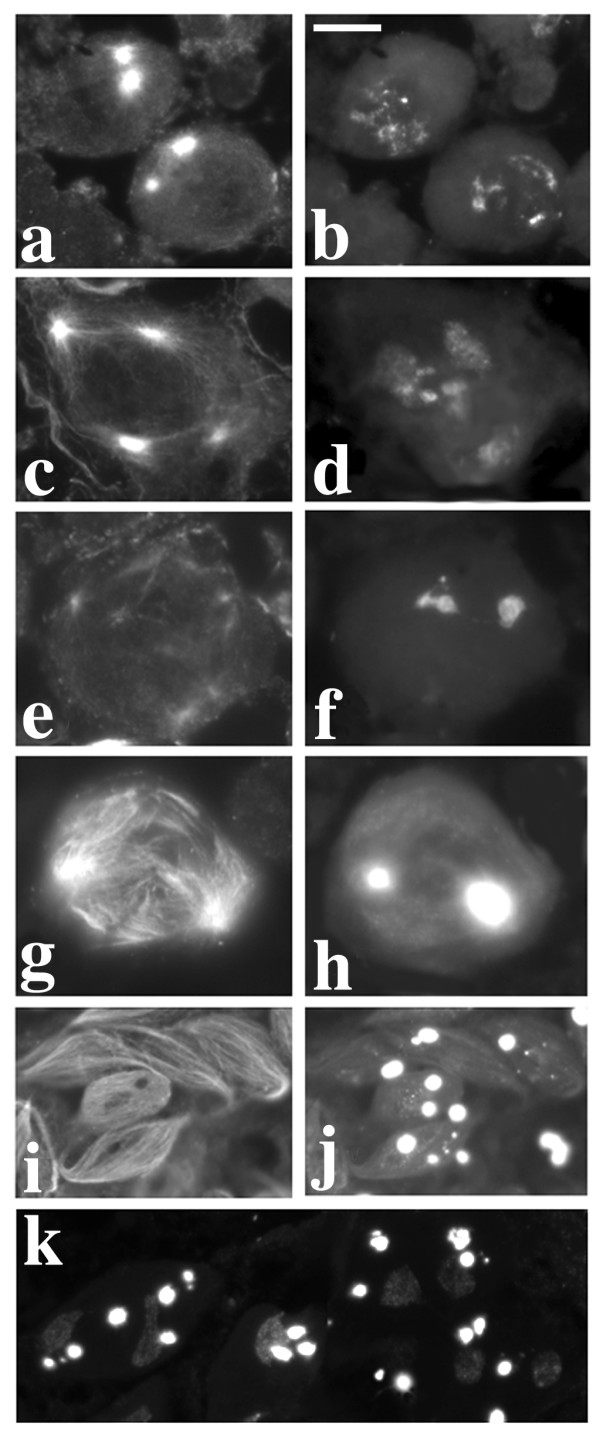
**Meiotic phenotype of *ms(3)162-39***. Left column: immunostaining with anti α-tubulin antibody; right column: Hoechst 33258 staining. For comparison, see the wild type meiosis reported in Figure 4. a-b: prometaphase nuclei in *ms(3)162-39 *primary spermatocytes. Note that asters are migrating to the opposite poles (a) while chromatin fails to compact (b). c-d: asters further separating in preparation for meiosis II (c), while chromatin is still undercondensed (d). e-f: chromatin reaches a level of compaction similar to wild type (f), but spindle fibers have lost their organization. g-h: anaphase figure showing defective chromatin segregation and a highly disorganized central spindle. i-j: abnormal spermatids during the elongation stage; note the abnormal microtubules organization (i) and the irregular size of nuclei. k: micro- and macro-nuclei associated with irregularly sized nebenkerns. Bar: 10 μm

**Figure 7:**
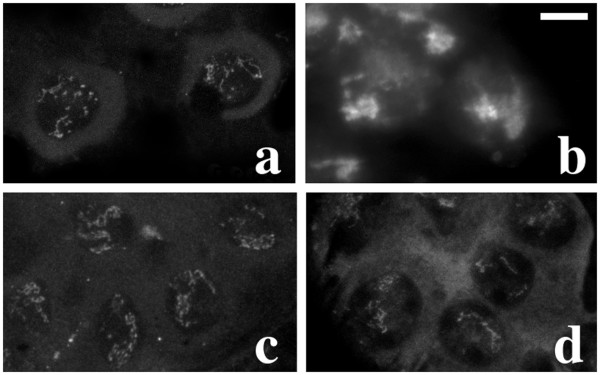
**Primary spermatocytes of mutants from Ceprani collection**. a-b: primary spermatocytes of *ms(2)Fra-1 *mutant males immunostained with either T53-1 (a) or S5 (b) antibodies; note that kl-3 loop is fragmented (a), ks-1 (faint fluorescence) is larger and kl-5 loop (bright fluorescence) is smaller than wild type (b); c-d: immunostaining with the T53-1 antibody of *ms(2)Fra-4 *(c) and *ms(2)Fra-15 *(d) mutant males; both show a fragmented kl-3 loop, which in *ms(2)Fra-15 *testes is also reduced. Bar: 10 μm

The cytological phenotypes of mutants are summarized as follows: Figure 1 (Hackstein collection), Figure 2 (Wasserman collection), Figure 3, Figure 4, Figure 5, Figure 6 (Zuker collection and wild type meiosis) and Figure 7 (Ceprani collection).

### Mutations with pleiotropic effects: meiosis failure

In this screening we isolated 5 lines in which primary spermatocytes are not able to carry out a normal meiosis and produce diploid spermatids, similar to the phenotype described for *twine *(*twe*) mutants [41].

The *ms(3)150-16 *(Figure 3e-3f) and *ms(3)167-72 *mutant lines (Figure 3g-3h) show a reduced and fragmented kl-3 loop while both kl-5 and ks-1 loops appear normal; the *ms(3)165-104 *mutant line shows a variable reduction and fragmentation of all loops (data not shown); the *ms(2)Fra-1 *mutant line exhibits a reduced and fragmented kl-3 loop (Figure 7a) while the other two loops are resized, with the kl-5 loop larger and the ks-1 loop smaller than in wild type (Figure 7b); in *ms(2)Fra-39 *the kl-3 loop is absent while the other two loops appear normal (data not shown).

The *ms(2)HA10 *mutation, which causes both a defective kl-3 loop and a degeneration of post-meiotic cells (Figure 1), is noteworthy since it is allelic to *ms(2)HA30*, which specifically affects the loops without producing other effects on meiosis (see loop-specific mutants section). Both alleles induce an extreme reduction of the kl-3 loop, compared to wild type (Figure 1g-1h). The *ms(2)HA10 *allele also induces a slight reduction of the kl-5 and ks-1 loops (data not shown) and meiosis failure, leading to the transformation of meiocytes into diploid pseudo-spermatids that initiate but fail to complete differentiation, resulting in cells which exhibit prominent sperm tails (Figure 1i-1j) which eventually degenerate. The gene specified by these mutations was mapped by standard recombination techniques to 2–86.8. Deficiency mapping, showed that *ms(2)HA10 *and *ms(2)HA30 *mutations are uncovered by *Df(2R)PC4 *but not by *Df(2R)P34 *and *Df(2R)Pcl-w5*, thus allowing us to locate the corresponding gene in polytene region 55D1-E6. *ms(2)HA10 *hemizygous males still exhibit a pleiotropic phenotype, while *ms(2)HA10/ms(2)HA30 *heterozygote males only display defects in the kl-3 loop and immotile sperm. Although we cannot completely rule out the possibility that the *ms(2)HA10 *line carries two closely associated male sterile mutations, our data suggest that the meiotic defects observed in *ms(2)HA10 *homozygous and hemizygous flies represent the pleiotropic effects of mutations at a single locus and that *ms(2)HA30 *represents a leaky mutation at this locus. According to previously described mutants from the same collection [42], we renamed the gene specified by the *ms(2)HA10 *and *ms(2)HA30 *mutations as *loop unfolding protein 3 *(*lup-3*).

### Mutations with pleiotropic effects: defective spermatids

We have identified 6 loci affecting both loop morphology and post-meiotic processes. The mutation *ms(2)04818*, subsequently renamed *double fault *(*dbf*), maps on the second chromosome, in polytene region 32A1-2 [36]. It has been described as a mutation affecting both the size of nuclei and nebenkern (mitochondrial derivatives) of onion-stage spermatids, and the shape of elongating spermatids [36]. Our analysis revealed the presence of an additional defect, a precocious disintegration of the kl-3 loop, which normally starts to grow in early primary spermatocytes, but then disappears before these cells are completely mature (data not shown).

*ms(3)168-112 *and *ms(3)127-109 *fail to complement, indicating that they are alleles of the same gene. Both these mutations induce a very strong reduction of the kl-3 loop (Figure 3a-3b) and a high frequency of irregular spermatids with variably sized nuclei and nebenkern. Staining of mutant testes with an anti α-tubulin antibody and with the DNA dye Hoechst 33258 revealed that cells undergoing meiotic divisions are frequently devoid of asters and exhibit hypercondensed chromatin (Figure 5) if compared to wild type (Figure 4). Notably, the number of nuclei inside each primary spermatocyte cyst appears normal in these mutants, indicating that the mutations specifically affect meiotic cells.

Males from *ms(3)162-39 *show a defect in both the ks-1 and kl-5 loops (Figure 3i-3j), but not in the kl-3 loop (data not shown). The kl-5 loop reacts only weakly with the S5 antibody, exhibiting a very weak staining pattern when compared to wild type. Moreover, the ks-1 loop is frequently either absent or strongly reduced and fragmented. In addition, spermatids are defective with large nebenkern associated with micro- and macro-nuclei (Figure 6), a phenotype suggesting failure in both chromosome segregation and cytokinesis [43]. We mapped *ms(3)162-39 *to the polytene region 85D8-E13, since it is uncovered by *Df(3R)by10*. *ms(3)162-39/Df(3R)by10 *hemizygous males show the same alterations observed in homozygous flies, strongly suggesting that *ms(3)162-39 *is a null allele.

Mutant males of I-induced *ms(2)Fra-4*, *ms(2)Fra-15 *and *ms(2)Fra-38 *lines show a defective kl-3 loop (Figure 7), while the morphology of the other loops is normal. Both *ms(2)Fra-4 *and *ms(2)Fra-15 *mutants show a fragmented kl-3 loop, the presence of defective spermatids with micro- and macro-nuclei and irregular nuclei-nebenkern associations (data not shown). In *ms(2)Fra-38 *testes, the kl-3 loop is often absent or strongly reduced, and irregular nuclei-nebenkern associations are observed (data not shown).

### Loop-specific mutations

We have identified two new genes, which specifically alter loop shape and development, without affecting other aspects of spermatogenesis besides sperm motility.

Three loop-specific mutations had been previously isolated from Hackstein collection, identifying two genes required for kl-3 loop unfolding [42]. Here we report the identification and characterization of an additional mutant line from this collection, *ms(2)HB108*, which causes a strong reduction of the kl-3 loop (Figure 1e-1f). Mutation *ms(2)HB108 *has been mapped by recombination at position 2–46.6. Consistent with the nomenclature of mutants from the same collection [42], we propose to rename *ms(2)HB108 *as *loop unfolding protein 4 (lup-4)*.

*ms(3)03817 *was isolated from the Wasserman collection of male steriles [36]. In this line, primary spermatocytes appear normal *in vivo *and no other abnormalities are observed in post-meiotic stages, except for immotile sperm tails. However, immunostaining with the S5 monoclonal antibody revealed that in almost 10% of primary spermatocytes either the kl-5 or the ks-1 loop is split in two identical halves. Moreover, in ~1% of spermatocytes both loops are split (Figure 2 and Table 3). This phenotype was never detected in wild type and was previously described only by Bonaccorsi and coworkers [19] in males carrying a rearranged Y chromosome. Because of this phenotype, we propose to rename the mutant as *double loops of the Y-1 *(*dolly-1*).

**Table 3:** Cytological characterization of kl-5 and ks-1 loops in *dolly-1 *mutants

	**Regular**	**3 loops**	**4 loops**
**Total**	494	46	4
**%**	90.80	8.45	0.75

Ten testes from *dolly-1 *males were immunostained with the S5 antibody. Aberrant primary spermatocytes were found in every preparation. Regular: nuclei containing one kl-5 and one ks-1 loop. 3 loops: either kl-5 or ks-1 loop is split. 4 loops: both kl-5 and ks-1 loops are split. kl-5 and ks-1 loops are equally affected.

Finally, line *ms(3)142-46 *shows an overall reduction of the kl-3 loop. Interestingly, in 70% of primary spermatocytes this loop is also split into two halves (Figure 3c-3d), but no defects are visible in germ line other than immotile sperm. This phenotype resembles that of the *dolly-1 *mutant described above. For this reason we propose to rename this mutant as *double loops of the Y*-2 (*dolly-2*).

## Discussion

### Analysis of mutations with pleiotropic effects

A large fraction of male sterile mutations analyzed in the present work greatly affect meiosis in the male germ line. We have isolated 13 loop-defective mutations also showing meiotic abnormalities. Six mutations have primary spermatocytes that do not undergo meiosis, but develop as spermatids with immotile sperm tails, a phenotype reminiscent of *twe *mutants [41]. Seven mutations exhibit a defective meiosis leading to the formation of aberrant spermatids. One important issue for these pleiotropic mutations is to understand whether loop abnormalities in these lines represent a specific effect of the mutation or only a side effect of cellular stress.

In the mutants with meiosis failure, we found that the kl-3 loop is either absent or abnormal and sometimes also the other two loops are reduced. The simplest interpretation of loop phenotypes in mutants with a meiotic failure is that defects impairing meiosis in primary spermatocytes also affect loop development, a process normally taking place in this cell type. If this were true, we would expect that all mutations with this phenotype should also share similar loop abnormalities. This is not the case as most of mutants with meiotic failure exhibit distinct loop phenotypes. In addition, we found that in *ms(2)Fra-34*, despite the meiosis failure, all loops appear completely normal by both phase contrast and immunohistochemical analysis (data not shown). Thus, we favor the idea that loop morphology and meiotic progression are separable events, and the effect on loop is not simply a by-product of meiosis failure.

Wakimoto and coworkers [39] demonstrated that in the Zuker collection of mutations affecting male fertility, about 20% of lines show a phenotype which can be explained by alterations in chromosome segregation at meiosis and/or aberrant cytokinesis, leading to defective spermatids. Also in this case we favor the hypothesis that loop defects represent specific effects of these mutations, and not a consequence of any alteration of chromatin behavior. Indeed, we have analyzed a number of male sterile lines with comparable defects in spermatids, such as *fusolo *[44], and they did not show abnormalities in loop morphology. Furthermore, three mutations, *ms(3)168-112*/*ms(3)127-109 *and *ms(3)162-39*, display irregular spermatids due to opposite defects in the timing of chromatin condensation and aster formation. In *ms(3)168-112*/*ms(3)127-109 *only the kl-3 loop is defective, while in *ms(3)162-39*, the kl-5 and ks-1 loops are affected but the kl-3 loop is normal. Also in this case we were unable to recover a generalized defect on loops.

### Analysis of loop-specific mutations

Six genes specifically affecting loop shape and development have been isolated in our previous [42] and present work (Table 2). Based on our immunohistochemical analysis, we can distinguish two kinds of alterations: either a substantial reduction/absence (*lup-1, lup-2, lup-3 *and *lup-4*), or a splitting of the loops (*dolly-1 *and *dolly-2*). Of these, only *dolly-1 *affects both the kl-5 and ks-1 loops, while all the others affect the kl-3 loop. At the moment we have no molecular data that can help us to understand the functions of these genes. Goldstein and coworkers [45] reported that the artificial deletion of either *kl-3 *or *kl-5 *loci by X-Y translocations leads to the absence of two high molecular weight polypeptides and to the simultaneous absence of the outer dynein arms of the peripheral doublets of the sperm axoneme. This led the authors to the conclusion that probably *kl-3 *and *kl-5 *loci harbor the genes encoding for the dynein subunits of the sperm axonemes. However, in both *lup-1 *and *lup-2 *mutant lines, that display reduced or missing kl-3 loop, these high molecular weight proteins are regularly synthesized, but were found to be unstable [42]. The *ms(2)HA30 *mutation at the *lup-4 *locus exhibits a cytological phenotype comparable to *lup-1 *and *lup-2 *mutants, but both the electrophoretic pattern and the outer dynein arms of sperm axonemes appear normal (C. Mencarelli, personal communication). Therefore, the kl-3 loop reduction and the associated male sterility observed at least in the *ms(2)HA30 *allele of the *lup-4 *complementation group, suggest that this loop might play additional roles besides harboring the coding sequence of a dynein subunit [46] and contributing in the stabilization of the peptides found to be unstable in *lup-1 *and *lup-2 *mutants [42].

One important function required for normal loop morphology is their stabilization. Loops have to initiate and maintain an unfolded state until they complete their function. In *lup-4 *and *ms(2)04818 *mutants it is possible that the main defect is a precocious disintegration of the loop, rather than a growth defect. A stabilization problem might also explain the defects in *dolly-1 *and *dolly-2 *mutants. In wild type, each loop is a single cytological structure formed by each sister chromatid, while in *dolly-1 *and *dolly-2 *loops are split in two identical moieties. We think that in these mutants, the two loops formed by each sister chromatid become independent and separate, although we cannot establish whether this separation is restricted to the Y loop-forming regions or it involves longer regions. In any case, if correct, this interpretation would suggest the existence of a mechanism required for the maintenance of loop integrity as a unit, whose failure would eventually cause sperm immotility, as observed.

We failed to recover mutations affecting the entire set of loops, except some causing a complete meiosis failure. We cannot rule out the possibility that genes controlling the development of all loops might also have strong pleiotropic effects compromising primary spermatocyte development, thus being excluded by our screening criteria. Nevertheless, our data are in agreement with the existence of distinct pathways regulating the morphogenesis of either kl-3 or kl-5/ks-1 loops. Most of the mutations isolated so far affect the kl-3 loop. We isolated only 3 mutations affecting loops kl-5 and/or ks-1. We suggest two ways to explain the relative abundance of kl-3 loop mutants: (i) a lower frequency of mutations affecting kl-5 and ks-1 loops, possibly because fewer genes are required for their control; (ii) the screening limitations represented by the *in vivo *preliminary analysis performed on some mutant collections. Indeed, it must be taken into account that in non-fixed testes preparations of *D. melanogaster *only the kl-3 loop is clearly detectable within the primary spermatocyte nuclei. On the other hand, mutations mildly affecting the size and/or the morphology of either the kl-5 or the ks-1 loop are very difficult to be identified by *in vivo *analysis alone. In this respect, it is noteworthy that the Hackstein collection, composed of lines that were pre-selected by *in vivo *analysis, only provided kl-3 loop defective mutants, while the other collections, which were entirely tested by immunostaining, also provided mutants affecting the other two loops.

It is also noteworthy that in lines *dolly-1, ms(3)162-39 *and *ms(2)Fra-1 *both the kl-5 and the ks-1loops look abnormal. This is not surprising since these two loops share a number of features: their DNA content is very similar [47-49], their transcripts behave similarly [50], their development is coupled [19] and they share some loop-binding proteins [19,22,23,32]. Therefore, it is likely that they are controlled, at least in part, by the same biochemical mechanisms.

Finally, it is interesting to note that loops are always immunostained by the S5 and T53-1 antibodies, even when they appear highly defective or strongly reduced. This indicates that the defective loop structure is still able to bind proteins and possibly perform some limited activity, that is however insufficient to grant male fertility. This view is supported by the previous discovery that *lup-1 *and *lup-2 *mutations affect the stability, but not the synthesis, of high molecular weight polypeptides probably involved in sperm axoneme formation [42].

### Considerations about the criteria used in the screening

It is known that male sterile mutations may either identify alleles of essential genes (identified by lethal alleles), or germ line specific genes. According to Wakimoto and coworkers [39], who made an estimation based on the screening of the Zuker collection, both these categories identify at least 500 complementation groups, considering the whole *D. melanogaster *genome. It is accepted that the Zuker mutation collection has saturated the two major autosomes of *D. melanogaster *[38-40], with every male sterile locus on these chromosomes being hit many times during the mutagenesis. This implies that a screening of this collection should provide a number of mutant alleles proportional to the gene sequence length for any considered gene. Ideally, it should recover more than one mutant allele for each gene. However, it must be noted that this saturation condition has not been accomplished in our screening, since only in two cases we found more than one mutant allele per locus: *ms(3)168-112 *and *ms(3)127-109*, plus an allele of the *lup-1 *gene, *ms(3)155-34 *(Table 2) [42]. Since the Zuker collection has been previously demonstrated to be saturated, and we exclude a bias in the size of the genes involved in loops development and/or control, we propose that the failure to recover more mutant alleles per locus is due to an intrinsic limitation of our screening criteria. As noted in the Results section, no mutations inducing degeneration of most germ cells were selected; however, it is possible that some of them might also have a role in loops behavior. In addition, male sterile lines showing weak loop defects were not included in the Results section. Also in this case, it is possible that we did not consider some lines representing alleles of the genes that we identified, as well as other genes that were not identified here. Interestingly, with the exception of the *dolly-1 *mutation, all the lines that we isolated display more than 70% defective spermatocytes, so they can be considered 'strong' alleles with regards to the loop phenotype. The inability to find multiple mutant alleles for each locus is reasonably due to the relatively high threshold of the criteria chosen in our screening. Nonetheless, additional alleles can be found among the two discarded categories of lines, by means of complementation tests.

## Conclusion

In the present work we carried out a large scale screening of 726 male sterile mutant lines from different sources. We analyzed them by immunofluorescence using antibodies directed against Y-loop-associated proteins. The screening allowed us to isolate several mutations showing defective Y-loops. In 8 lines this is the only phenotype detected, in 14 lines the abnormal loop phenotype is associated to additional defects in male spermatogenesis (Table 1). Our data suggest that, in most cases, the pleiotropic defects in male germ line are not a consequence of cellular stress, but that they are directly or indirectly correlated to loops abnormalities. We believe that the molecular characterization of the genes identified by the mutations described here will provide new insights into the long-lasting debate concerning the biological role of *Drosophila melanogaster *Y chromosome loops and a through elucidation of a mechanism involving heterochromatin regulation in eukaryotes.

## Methods

### Drosophila stocks

All stocks used in the present work were reared at 25°C on standard cornmeal medium. All mutations are maintained in stable stocks using multiple inverted, balancer chromosomes; we used *CyO *for mutations on the second chromosome and *TM6C *for mutations on the third chromosome. For further details regarding balancers see [51]. The deletions we used for cytological mapping were obtained from the Umeå Stock Center and cover ~80% of *Drosophila *chromosomes 2 and 3. When necessary, deletions have been rebalanced over *TM6C *chromosome and then crossed with mutant stocks.

### Testis immunofluorescence

Larval, pupal and adult testes were dissected and fixed according to Pisano and coworkers [29]. Slides were then rinsed twice in Dulbecco's PBS modified formula for 5 minutes and incubated for 1 hour in humid chamber at room temperature with 20 μl of either the T53-1 antiserum [29] diluted 1:10 in PBS, or the S5 monoclonal antibody [32] diluted 1:20 in PBS. After washing in PBS, slides were incubated for one hour with the secondary antibody, a sheep anti-mouse IgG conjugated with fluorescein (FLUOS, Boehringer), diluted 1:20 in PBS. Finally, slides were air dried and mounted in PBS containing 0.5 μg/ml Hoechst 33258 dye. Immunofluorescence of microtubules was performed as described in [52] using a commercial monoclonal anti α-tubulin primary antibody (Amersham) diluted 1:50 in PBS; the secondary antibody is an anti-mouse FITC. All cell stages were identified as described in [52].

### Light microscopy

Microscope analysis and pictures were made using a Zeiss III RS photomicroscope equipped with an HBO fluorescent light (100 Watts), or with a Zeiss Axioplan photomicroscope equipped with an HBO fluorescent light (50 Watts). We used Zeiss filter combination 09 for immunostained preparations with FLUOS-conjugated secondary antibodies, and Zeiss filter combination 01 for the Hoechst 33258 staining. Pictures at the Zeiss Axioplan microscope were taken using a CCD camera from Photometrics and saved using IP Lab Spectrum^® ^software. Composite pictures were prepared using Adobe Photoshop^®^.

## Authors' contributions

FC performed the I-R hybrid dysgenesis, analyzed the Ceprani collection, made the complementation tests and prepared the corresponding picture; GDF participated in the screening of the Zuker collection, made the crosses for the complementation tests and mapping, prepared the corresponding pictures, and contributed to the manuscript preparation; RPe participated in the screening of the Zuker collection; RPi screened the Hackstein and Wasserman collections, participated in the screening of the Zuker collection, made the complementation tests, and prepared the manuscript and tables. All authors read and approved the final manuscript.
